# Dysregulation of mCD46 and sCD46 contribute to the pathogenesis of bullous pemphigoid

**DOI:** 10.1038/s41598-017-00235-3

**Published:** 2017-03-10

**Authors:** Pei Qiao, Erle Dang, Tianyu Cao, Hui Fang, Jieyu Zhang, Hongjiang Qiao, Gang Wang

**Affiliations:** Department of Dermatology, Xijing Hospital, Fourth Military Medical University, Xi’an, China

## Abstract

Bullous pemphigoid (BP) is an autoimmune bullous disease caused by autoantibodies against BP180 in the epidermal basement membrane. Autoantibody-mediated complement activation is an important process in BP pathogenesis. CD46, a crucial complement regulatory protein in the complement activation, has been reported to be involved in several autoimmune diseases. In the present study, we investigated whether CD46 plays a role in BP development. We found that sCD46 expression was significantly increased in the serum and blister fluids of BP patients and correlated with the levels of anti-BP180 NC16A antibody and C3a. Otherwise, the level of mCD46 was decreased in lesions of BP patients, whereas the complement activation was enhanced. We also found that CD46 knockdown in HaCaT human keratinocytes enhanced autoantibody-mediated complement activation. Importantly, exogenous CD46 blocked complement activation in both healthy skin sections and keratinocytes induced by exposure to pathogenic antibodies from BP patients. These data suggest that CD46 deficiency is an important factor in BP pathogenesis and that increasing CD46 levels might be an effective treatment for BP.

## Introduction

Bullous pemphigoid (BP) is an autoimmune subepidermal blistering disease characterized by production of autoantibody directly responding to pathogenic antigen 180 (BP180) within the dermal-epidermal junction (DEJ)^1, 2^. The production of autoantibodies directed against the non-collagenous 16A domain (NC16A), which was the transmembrane domain of BP180, activated the complement system to initiate a series of inflammatory events, including dermal mast cell degranulation, generation of eosinophil-rich infiltrates and subsequent blister formation^3^. Autoantibodies from BP patients can bind complement *in vitro* and the main component of the complement system C3b can be detected at the basement membrane zone (BMZ) of the lesional skin by direct immunofluorescence (IF)^4^. In a passive transfer BP mouse model, pathogenesis following injection with BP autoantibodies was delayed in mice deficient in component C4 or factor B^5, 6^. These findings suggest that activation of the complement system is critical in BP development. However, the upstream regulators of this process are largely unknown.

Complement regulatory proteins (CRPs) are an important class of regulatory proteins in the complement system that control enzyme cascades, assembly of the membrane attack complex, and homeostasis of the complement system^7, 8^. Dysregulation of these proteins directly affects the progression of several autoimmune diseases. CD46 is a 44-kDa CRP that mainly exists in the membrane-bound form and is expressed by all cell types, with the exception of erythrocytes. CD46 mainly protects autologous cells from complement attack by inhibiting C3 inactivation^9, 10^. It can also bind to opsonins C3b and C4b and act as a cofactor in their proteolytic degradation through serine-protease factor I^11, 12^. It has been reported that CD46 expression and function are impaired in some autoimmune diseases. In systemic lupus erythematosus (SLE) patients, mCD46 expression was found to be downregulated during lymphopenia and neutropenia relative to that in healthy subjects, whereas disease severity was associated with activation of the complement system^13–15^. In addition, decreased CD46 expression was associated with aggravation of the clinical symptoms of rheumatoid arthritis^16–18^.

The presence of autoantibodies causes the classical complement system pathway to be activated, leading to deposition of C3b in the BMZ of BP lesions^19^. Given that CD46 controls complement activation by suppressing C3 activity, we hypothesized that loss of CD46 contributes to BP development. We found that CD46 knockdown in HaCaT human keratinocytes enhanced autoantibody-mediated complement activation, whereas overexpressing CD46 blocked this process. Our results demonstrate an inhibitory role of CD46 in BP progression and suggest that it might be a therapeutic target for BP.

## Results

### Elevated sCD46 in serum and blister fluids of BP patients

**Figure 1:**
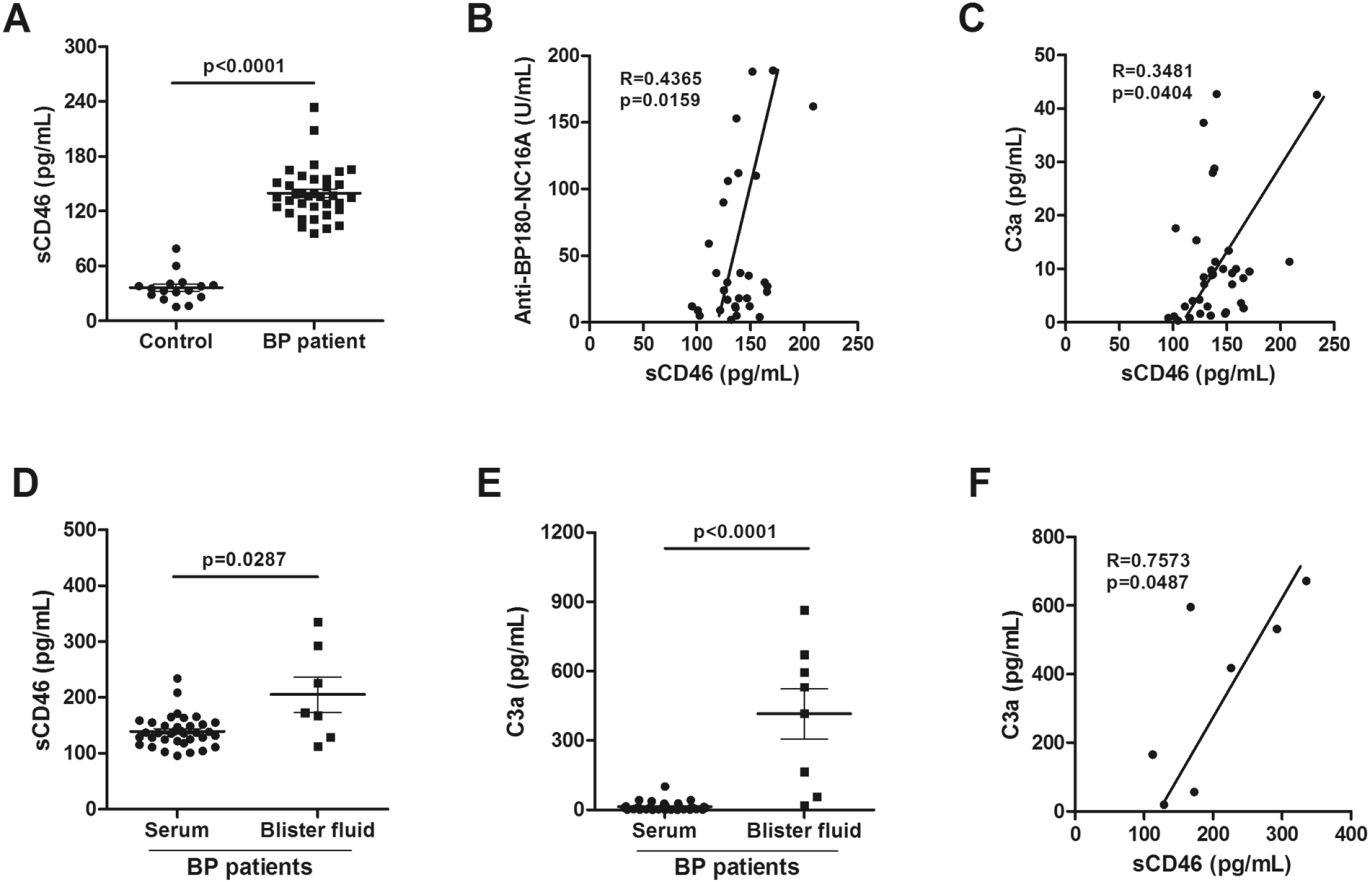
Up-regulation of sCD46 expression in serum and blisters of BP patients. (**A**) ELISA assay was used to determine the sCD46 level in serums from 36 BP patients and 16 healthy controls (**B**,**C**) Positive correlations for ELISA assay between the levels of (**B**) sCD46 and anti-BP180-NC16A titer and (**C**) sCD46 and C3a in serums of BP patients. (**D**,**E**) ELISA assay of protein levels of (**D**) sCD46 and (**E**) C3a in blister fluids from 7 BP patients. (**F**) Positive correlations for ELISA assay between the levels of sCD46 and C3a in blister fluids of BP patients. P < 0.05 was considered significant.

Normally, two types of CD46 exist in humans: mCD46 and shed sCD46. We first detected the expression of sCD46 in serum and blister fluids from BP patients by ELISA. The serum sCD46 concentration in 36 patients was 139.50 ± 28.21 pg/mL (Fig. 1A), which was significantly higher than that of 16 normal controls (36.26 ± 15.68 pg/mL). The level of sCD46 was correlated with the levels of anti-BP180 NC16A antibody (Fig. 1B) and C3a (Fig. 1C) respectively, which are biomarkers reflecting the activity and severity of BP. Additionally, the blister fluids from BP patients were found to have a much higher concentration of sCD46 (205.10 ± 83.51 pg/mL, n = 7) (Fig. 1D) and C3a (415.30 ± 307.4 ng/mL, n = 8) (Fig. 1E) than the serum. ELISA results from 3 BP patients’ serum and blister fluids were consistent with this finding (Fig. S1). We also found a significant positive association between the concentrations of sCD46 and C3a (Fig. 1F). These results indicate that sCD46 is up-regulated in both the serum and the blister fluids of BP patients and that its level was correlated with activity and severity of BP.

### Abnormal down-regulation of mCD46 in epidermal keratinocytes in BP patients

**Figure 2:**
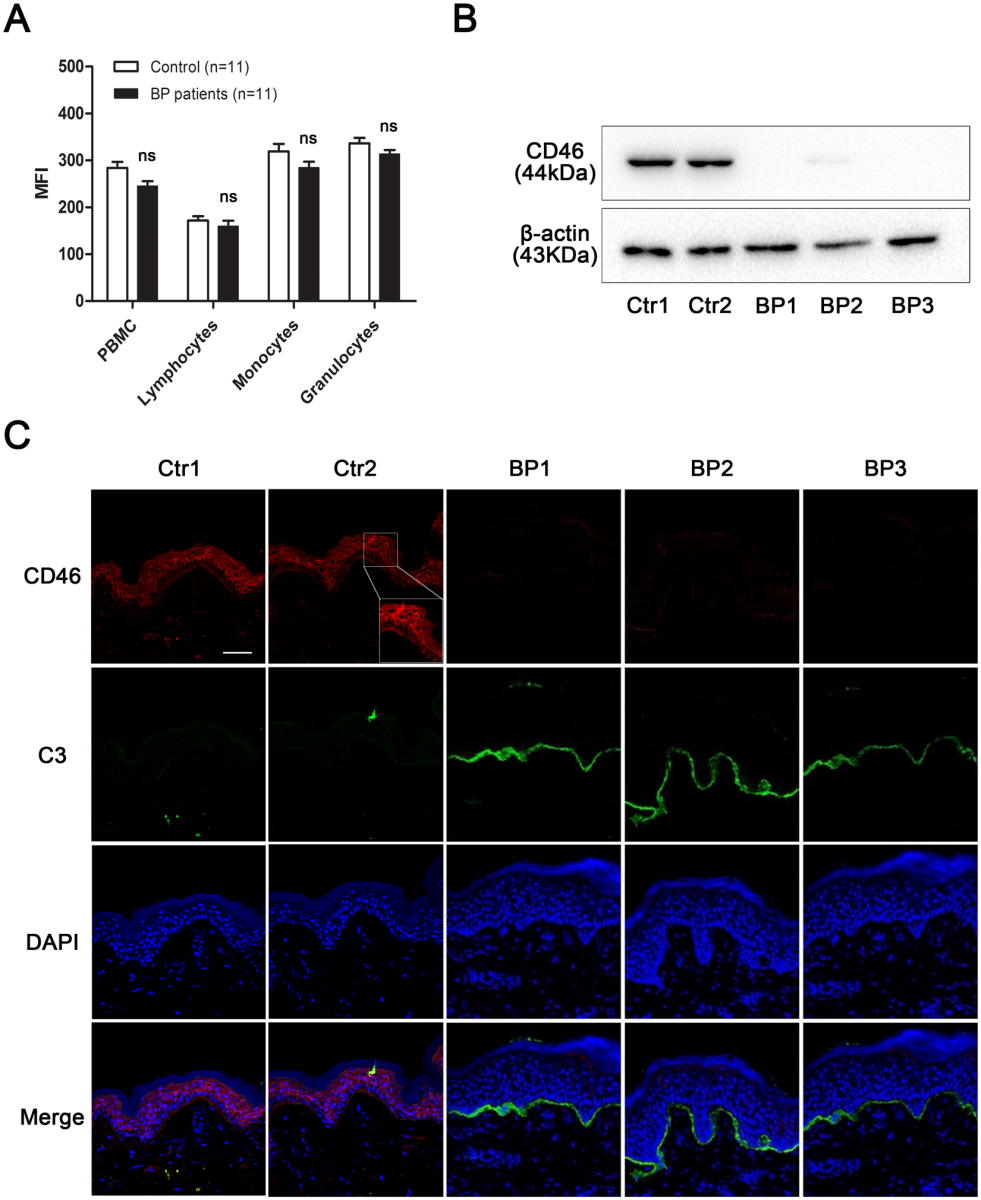
Down-regulation of mCD46 in BP patients. (**A**) Flow cytometry analysis of mCD46 expression in peripheral blood. Results represent the mean ± SEM from eleven independent experiments. NS represent no significant difference between control and BP patients. (**B**) Protein expression of CD46 in skin sections was examined by western blot. (**C**) IF staining was performed on normal skin sections and lesions from BP patients to measure CD46 expression in the epidermal keratinocytes and C3 deposition in the DEJ. DAPI staining of nuclei is shown in blue. Scale bar, 100 nm.

To confirm the expression of mCD46 in BP, PBMCs and lesional skin from BP patients were collected. Flow cytometry revealed a slight reduction of mCD46 in PBMCs, especially in monocytes and multinucleated cells, when compared with the levels in healthy controls (Fig. 2A). However, western blot analysis of skin extraction of 3 BP patients and 2 healthy controls revealed that CD46 was down-regulated in BP patients compared with that in healthy controls (Fig. 2B). To further verify this result, we performed IF staining on paraffin sections of samples from 10 BP patients. There have a high level of mCD46 in the stratum spinosum and the stratum granulosum in the epidermal and a moderate expression of mCD46 in the basal membrane of healthy control. However, weak staining for CD46 was observed in epidermal specimens from BP patients (Fig. 2C), which was consistent with the results of the western blot analysis. Taken together, these results indicate that mCD46 expression is abnormally down-regulated in the epidermis of BP patients and weak expression of mCD46 might responsible for the activation of complement system and deposition of C3 in the DEJ of BP patients.

### CD46 depletion enhances BP IgG-mediated complement activation ***in vitro***

**Figure 3:**
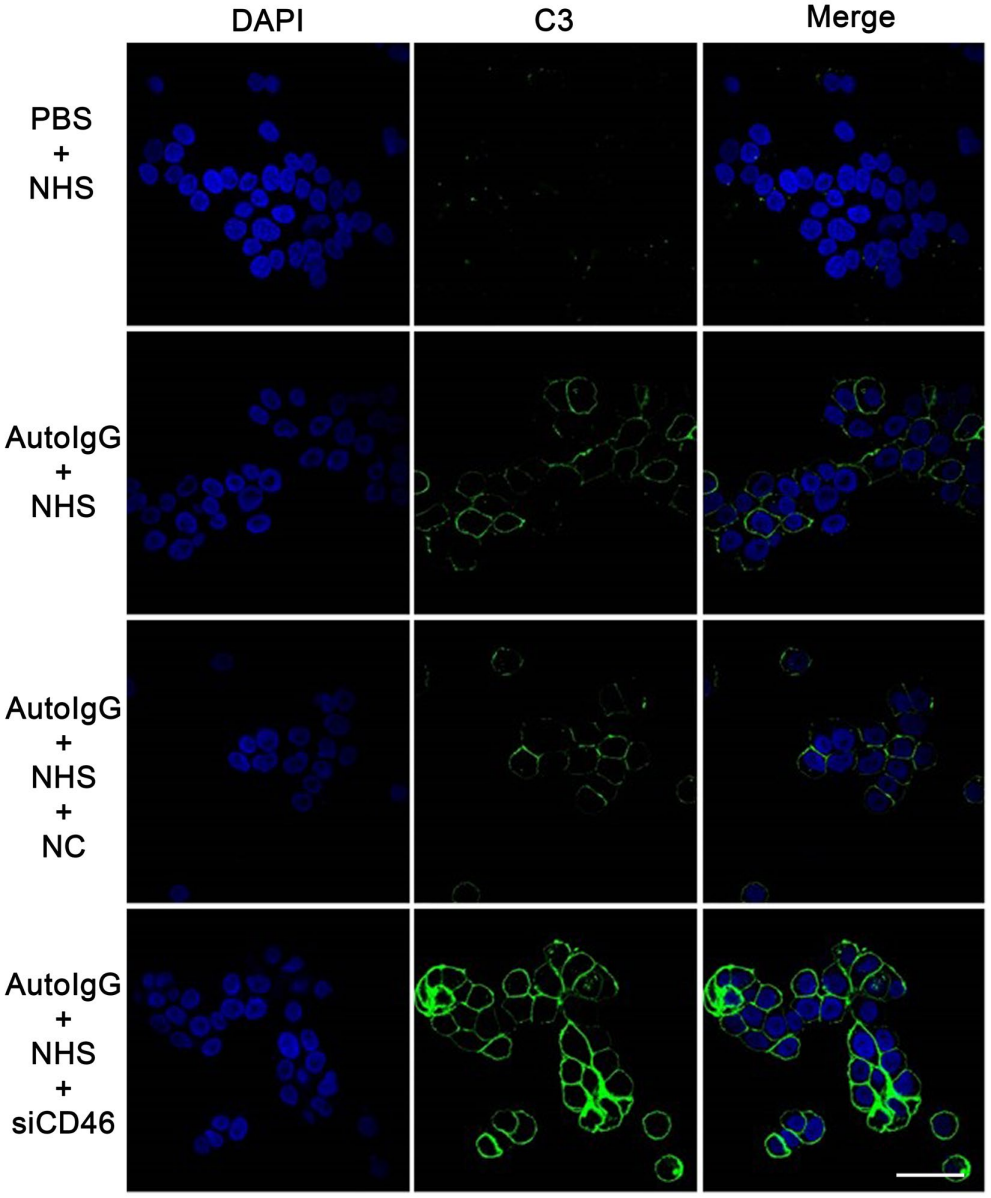
CD46 depletion enhances auto-IgG-mediated C3 deposition in HaCaT cells. HaCaT cells transfected with CD46 siRNA were incubated with purified auto-IgG overnight at 37 °C. Fresh healthy serum containing complement components was added to mimic BP pathogenesis. C3 deposition was detected by IF staining to determine the complement activation level. Scale bar, 25 nm. Auto-IgG, pathogenic IgG from BP patients’ serum; NHS, normal human serum; NC, negative control siRNA.

To investigate whether the abnormal expression of CD46 was involved in the pathogenesis of BP, HaCaT cells were treated with pathogenic IgG from BP patients and complement components from the serum of healthy controls respectively, and C3 deposition was evaluated 2 h later by IF staining. The effectiveness of CD46 siRNA and transfection system showed in Fig. S2. As expected, obvious C3 deposition was observed on the HaCaT cell membrane after incubation with pathogenic autoantibodies, and no signal was observed in the negative control treated with PBS. SiRNA-mediated downregulation of CD46 resulted in significantly increased C3 staining compared with both the positive and negative control (Fig. 3). These results indicate that CD46 deficiency leads to increased autoantibody-mediated C3 deposition in keratinocytes.

### Recombinant CD46 inhibits autoantibody-mediated C3 deposition in BP pathogenesis

**Figure 4:**
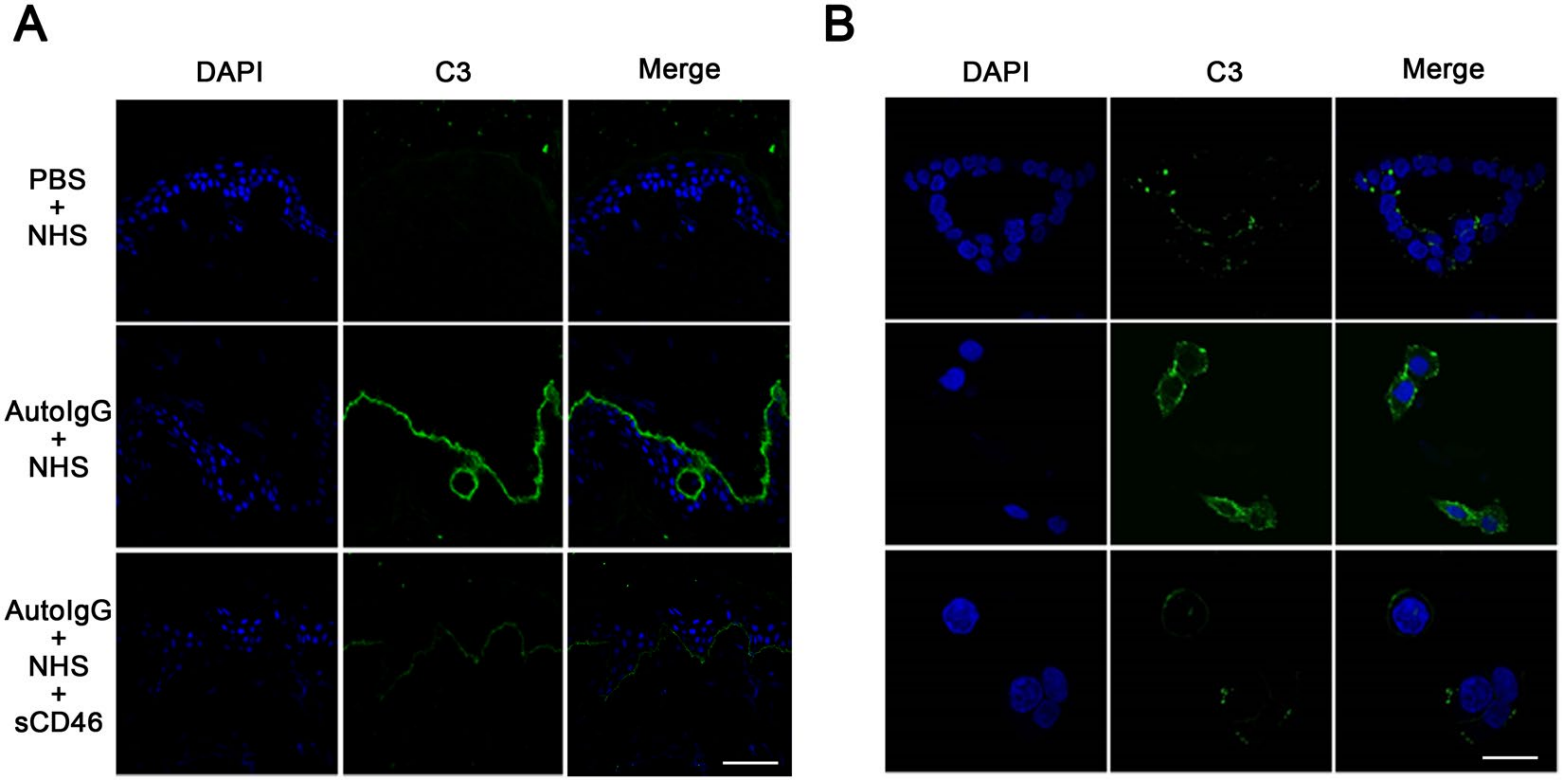
Recombinant CD46 inhibits autoantibody-mediated C3 deposition in BP pathogenesis. (**A**) Frozen skin sections from healthy controls and (**B**) HaCaT cells were incubated with purified auto-IgG and fresh serum from healthy controls containing complement components. C3 deposition was detected by IF staining to determine the complement activation level. Scale bars, 100 nm in (A) and 25 nm in (B) respectively. Auto-IgG, pathogenic IgG from BP patients’ serum; NHS, normal human serum; NC, negative control siRNA.

To clarify the mechanism by which CD46 mediates complement activation by BP patient autoantibodies, we incubated frozen skin sections from BP patients and HaCaT cells with pathogenic IgG and fresh serum from healthy controls containing complement components, and C3 deposition was evaluated 2 h later by IF staining. The result showed that C3 deposition was obviously expressed in both HaCaT cells and the BMZ of BP patient skin samples in the presence of pathogenic IgG and was inhibited by treatment with 10 μg/ml recombinant human CD46 protein (Fig. 4). These results suggest that CD46 suppresses the complement activation during BP pathogenesis.

### MMP9 and ADAM10 are responsible for shedding of mCD46 in BP pathogenesis

**Figure 5:**
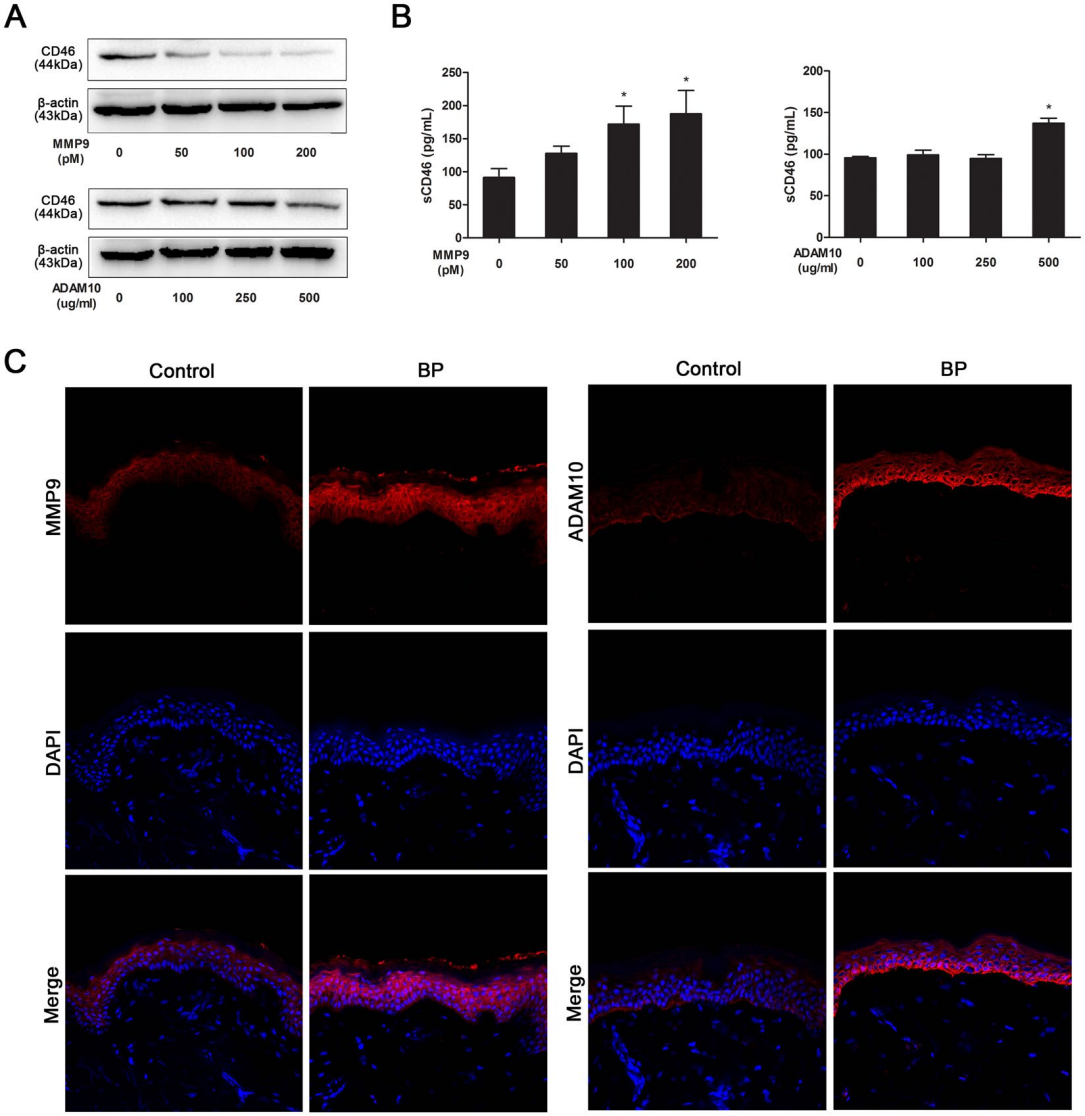
MMP9 and ADAM10 are responsible for shedding of mCD46 in BP pathogenesis. HaCaT cells were incubated with activated MMP9 (50, 100 and 200 pM) and ADAM10 (100, 250 and 500 ng/mL) proteinases at 37 °C for 2 h. (**A**) The HaCaT cells were collected and CD46 were detected by western blot after incubation. (**B**) Elisa assay were used to value the sCD46 in the supernatant that shedded by MMP9 and ADAM10. Results represent the mean ± SEM from three independent experiments. P < 0.05 was considered significant. (**C**) IF staining was performed on normal skin sections and lesions from BP patient measure MMP9 and ADAM10 expression in the epidermal keratinocytes. DAPI staining of nuclei is shown in blue. Scale bar, 100 nm.

As previous studied have reported that mCD46 could be shedded by matrix metalloproteinases (MMPs) and the disintegrin and metalloproteinase (ADAM) family, we incubated HaCaT cells with MMPs or ADAM to verify the shedding process. The results showed that mCD46 were significantly decreased in HaCaT cells after incubated with MMP9 or ADAM10 for 2 hours at 37 °C (Fig. 5A). The sCD46 that shedded by MMP9 increased in a dose-dependent manner in the cultural supernatant (Fig. 5B). Meanwhile, we confirmed that both MMP9 and ADAM10 increased in the epidermal from BP patients, when comparing with healthy controls (Fig. 5C). These results suggest that MMP9 and ADAM10 are involved in the BP pathogenesis via regulating the balance of mCD46 and sCD46.

## Discussion

As a critical cofactor in the inactivation of C3b and C4b, CD46 regulates immune responses mediated by autoantibodies^20^. CD46 and its role in certain diseases, including inflammatory diseases, allergy and autoimmune diseases, have attracted increasing attention over the past two decades. In the present study, we observed an elevated level of sCD46 both in the serum and blister fluids of BP patients, which was positively associated with disease severity. However, the level of mCD46 was decreased in peripheral blood cells, especially in the lesional skin of BP patients. Moreover, Depletion of CD46 enhanced C3 deposition and autoantibody-mediated complement activation, and CD46 protected host cells from autoantibody-mediated complement activation. These data prove that CD46 can function as an inhibitory capacity in the pathogenesis of BP.

BP is an autoimmune subepidermal blistering disease caused by IgG autoantibodies targeting the noncollagenous 16A (NC16A) domain of human collagen 17^21, 22^. Previous studies using a passive transfer mouse model demonstrated that these autoantibodies mediated the pathogenic mechanisms of BP, including autoantibody binding, complement fixation and activation, immune cell infiltration, proteinase secretion, and subepidermal blister formation. Complement activation is one of the pivotal steps in BP development. It has been shown that using the F(ab’)2 fragments of pathogenic IgG could block the autoantibody-mediated complement activation^23^. A recent study also demonstrated the inhibitory role of anti-NC16A IgG4 by blocking IgG1- and IgG3-induced complement fixation, indicating that complement activation is tightly regulated, even in pathogenic conditions^24^. However, the direct regulatory mechanism of complement activation is still not well elucidated in BP. Here, we found that the balance between membrane and soluble CD46 is disturbed in BP patients and that downregulation of mCD46 might play an important role in the excessive activation of the complement system in the BMZ of BP patients.

CD46 is a type I transmembrane glycoprotein that was initially discovered as an essential membrane-bound CRP^25^. It functions as a cofactor in the inactivation of C3b and C4b to prevent host cells from being destroyed by the immune system. CD46 is diffusely distributed in the human body and is expressed in all cells, except erythrocytes. In addition to mCD46, sCD46 has also been detected in several biological fluids, most notably in seminal plasma. Three forms of CD46 exist in normal individuals and cancer patients, with molecular masses of 56, 47, and 29 kDa. The 29 kDa sCD46 is considered to be released from the membrane by proteolytic cleavage of cell surface CD46, and the 47 kDa and 56 kDa sCD46 are produced by intron retention of mRNA, probably in the liver^26^. A previous study shows that sCD46 is elevated in the serum of patients with active SLE and its levels are correlated with complement activation, which suggests that CD46 can be used as a biomarker for certain autoimmune diseases^27, 28^. In this study, we observed up-regulation of sCD46 in the serum and blister fluid of BP patients and found that sCD46 levels were correlated with disease progression, which indicates that sCD46 can be used as a clinical biomarker for BP.

CD46 ectodomain is composed of four conserved short consensus repeats, which are also called sushi domains or complement control protein modules (CCP). CCP of CD46 helps to identify the deposited C3b and C4b and mediate their cleavage by serine protease factor I, thus protecting the cell from complement attack. Normally, surface expression of CD46 on a variety of cell types is tightly regulated. CD46 can be constitutively internalized via clathrin-coated pits and recycled to the cell surface^29^. Additionally, MMPs and the disintegrin and ADAM family are responsible for shedding of the ectodomain of CD46. It is well documented that sCD46 is not differentially regulated in the serum of SLE patients, but is down-regulated in certain cells involved in disease development. Our study confirm the disturbed balance between sCD46 and mCD46 by MMP9 and ADAM10 in BP patients. Considering that MMPs and ADAMs, which are related with the BP development, cause the shedding of the BP180 ectodomain, we speculate that MMPs and ADAMs could also cause shedding of mCD46, weaken the complement regulatory system, enhance complement activation, and promote the blister formation.

Application of recombinant CD46 has been shown to inhibit immune complex-mediated inflammation in certain disease models. Several studies have validated the therapeutic efficacy of recombinant sCR1, a short consensus repeats sCR1 in CD46 molecular, in various autoimmune and inflammatory disorders^30–32^. Complement-based therapeutics have been considered for the treatment of BP^33–35^. A genetically engineered Fab antibody and scfv against BP180-NC16A IgG subtype without complement activation were reported to have potential therapeutic value in BP^36^. However, application of complement regulation as a therapeutic strategy has not been studied in BP. In this study, we identified an inhibitory role of CD46 in autoantibody-mediated complement activation, suggesting that CD46 might be used as a potential therapeutic molecule in BP or an alternative strategy when patients have been cleared of all clinical signs upon classical treatment. However, further studies are still needed to confirm its therapeutic function in BP mouse models.

Taken together, our findings demonstrate that CD46 is a key inhibitor of complement activation whose downregulation may be involved in the pathogenesis of BP and whose replenishment might be a potential therapeutic strategy for BP.

## Methods

### Human blood and skin samples

Blood and skin samples were obtained from 36 newly diagnosed BP patients (15 male and 21 female; age range: 45–81) (Table S1). The patients were diagnosed based on clinical symptoms and histological examination of skin samples, and had not received any systemic or local therapy for at least 2 weeks before skin biopsies were obtained from lesions. The normal control group included 16 healthy volunteers (7 male and 9 female; age range: 36–75) who had no history of autoimmune diseases. The study protocol was designed and carried out according to the principles of the Declaration of Helsinki and was approved by the ethics review board of the Fourth Military Medical University. Written informed consent was obtained from all participants prior to the study.

### ELISA

Blood and blister fluids were collected for ELISA to determine the level of anti-BP180 NC16A antibody, C3a and sCD46. The samples were tested using an antiCOL17 ELISA kit (MBL, Nagoya, Japan), C3a and sCD46 ELISA Development kits (Senxiong Biotech, Shanghai, China) according to the manufacturer’s instructions. Absorbance was read at 450 nm.

### Cell culture and CD46 interference

HaCaT human keratinocytes were cultured in Dulbecco’s Modified Eagle Medium (DMEM) containing 1.05 mM calcium chloride (Gibco-Invitrogen, Carlsbad, CA, USA) and supplemented with 10% fetal bovine serum (Gibco-Invitrogen) in a humidified atmosphere of 5% CO_2_ at 37 °C. After 24 h of serum starvation, cells at 60% confluence were transfected with short interfering siRNA targeting CD46 mRNA or negative control siRNA (100 nM; Ribibio, Guangzhou, China) using Lipofectamine 3000 reagent (Invitrogen) according to the manufacturer’s instructions, and then harvested 48 h later.

### *In vitro* complement activation

Auto immunoglobulin IgG was purified from 15 ml of mixed serum from BP patients using HiTrap Protein G and a HiTrap N-hydroxy-succinimide-activated high-performance affinity column (Amersham Biosciences, Little Chalfont, UK) coated with the BP180 non-collagenous 16A domain (NC16A). HaCaT cells seeded on coverslips in 6-well plates were incubated overnight with 1 μg/ml purified pathogenic IgG from BP patients, followed by addition of 10 μg/ml recombinant CD46 protein and 1 ml of fresh serum and incubation for 2 h to initiate complement activation to simulate the BP phenotype. C3 deposition at the DEJ and cell membrane was used as a measure of the degree of complement activation.

### Cell culture and proteases shedding

HaCaT human keratinocytes were cultured as previously described. Cells were seeded on coverslips in 6-well plates and at a confluency of approximately 70–80%. Human MMP9 (Abcam; ab157344) was activated by incubating with 2 mM 4-aminophenylmercuric acetate (APMA; Sigma) at a 9:1 (MMP9:APMA) ratio for 2 h at 37 °C. After 12 h of FBS starvation in DMEM, cells were stimulated with MMP9 and ADAM10 (0.1 ug/ml, 0.25 ug/ml, 0.5 ug/ml) for 3 h at 37 °C, respectively. The supernatant was used for ELISA and cells were homogenized in cell lysis reagent for western blot analysis.

### Western blot analysis

To obtain total protein from skin samples, the tissue was ground in liquid nitrogen and lysed in lysis buffer, and constituent proteins were denatured in sodium dodecyl sulfate (SDS). Following centrifugation at 10,000 rpm for 15 min at 4 °C, the total extracted proteins (30 mg) were separated by 10% SDS-polyacrylamide gel electrophoresis and transferred to a polyvinylidene difluoride membrane (Invitrogen). After blocking for 2 h with blocking buffer (Tris-buffered saline containing 5% skim milk and 0.01% Tween-20), the membrane was incubated overnight at 4 °C with antibodies against CD46 (Abcam, Cambridge, MA, USA) and β-actin (Abcam), washed with phosphate-buffered saline (PBS), and then incubated with horseradish peroxidase (HRP)-conjugated goat anti-rabbit secondary antibodies (Dako) for 1 h at room temperature. Immunoreactivity was visualized by enhanced chemiluminescence. CD46 expression levels were quantitated using the AlphaImager Gel Imaging System (Alpha Innotech, San Leandro, CA, USA).

### IF and confocal microscopy

HaCaT cells were seeded on coverslips in 6-well plates and then treated with BP auto-IgG and fresh serum, as described above. The coverslips were rinsed twice with 0.01 mM PBS and fixed in freshly prepared 4% paraformaldehyde for 15 min, followed by treatment with 0.3% Triton X-100 for 10 min at room temperature. Non-specific binding was blocked with 4% bovine serum albumin (BSA) at 37 °C for 40 min. After rinsing three times with 0.01 mM PBS, cells were incubated with a rabbit anti-human CD46 monoclonal antibody (1:200; Abcam) overnight at 4 °C, followed by a Cy3-labeled goat anti-rabbit IgG for 1 h in the dark at 37 °C. Nuclei were visualized by staining with 4′-6-diamidino-2-phenylindole (DAPI). Samples were analyzed by confocal microscopy (FV-1000/ES; Olympus, Tokyo, Japan). Tissue sections from BP patients and healthy controls were treated with anti-CD46, anti-MMP9 or anti-ADAM10 antibody overnight at 4 °C as described above, and then incubated with Cy3-labeled goat anti-rabbit IgG and DAPI. Images were acquired with a confocal microscope (Olympus Corporation, Tokyo, Japan). Treated HaCaT cells and tissues were rinsed twice with PBS and blocked with 4% BSA for 40 min. The cells were labeled with a fluorescein isothiocyanate (FITC)-conjugated anti-C3 antibody (1: 200; MP Biomedicals, Solon, OH, USA), followed by an HRP-conjugated goat anti-rabbit secondary antibody (1:500; Invitrogen). After three washes, the samples were incubated with DAPI and visualized by confocal microscopy.

### Flow cytometry analysis of cell membrane CD46 expression

To label lymphocytes, monocytes, and multinucleated cells, 100 μl of blood diluted in PBS to 10,000 red blood cells/μl was added to polystyrene tubes (BD Biosciences, San Diego, CA, USA) and stained with 8 μl/tube of FITC-conjugated monoclonal antibody against CD46 (BD Biosciences). After a 20 min incubation at room temperature, samples were resuspended in 0.5 ml of PBS and analyzed by flow cytometry.

### Statistical analysis

Statistical analyses were performed using GraphPad Prism v.4.0 software (GraphPad Inc., San Diego, CA). All the results were showed as mean ± SEM. Differences between groups were compared using Mann-Whitney test; Spearman’s rank correlation was used for correlation analyses. Differences with P < 0.05 were considered statistically significant.

## Electronic supplementary material

Supplementary Information
