# Supplementary material for: Dysregulation of mCD46 and sCD46 contribute to the pathogenesis of bullous pemphigoid
Source: Sci Rep. 2017 Mar 10;7:145. doi: 10.1038/s41598-017-00235-3 (PMC5428046; doi:10.1038/s41598-017-00235-3)
Supplement: Supplementary file 1 — Supplementary Information [file 41598_2017_235_MOESM1_ESM.pdf]

Supplementary information

Dysregulation of mCD46 and sCD46 contribute to the pathogenesis of bullous pemphigoid

Pei Qiao[1, 2], Erle Dang[1, 2], Tianyu Cao[1], Hui Fang[1], Jieyu Zhang[1], Hongjiang Qiao[1] and Gang Wang[1, *]

[1] Department of Dermatology, Xijing Hospital, Fourth Military Medical University, Xi'an, China

[2] These authors equally contributed to this paper

*Corresponding author: Department of Dermatology, Xijing Hospital, Fourth Military Medical University, Xi'an, Shaanxi Province, China. Tel. & fax: +86-29-84775401.

E-mail address: xjwgang@fmmu.edu.cn (Gang Wang)

Supplementary Table

Table S1. Demographic Characteristics of BP Patients

Bullous Pemphigoid Number	Sex	Age	Anti-BP180-NC16A (U/ml)	Bullous Pemphigoid Number	Sex	Age	Anti-BP180-NC16A (U/ml)
B114	Female	40	9	B378	Female	51	18
B128	Female	54	106	B379	Male	62	110
B141	Male	67	-	B382	Male	79	162
B144	Female	70	37	B383	Female	46	188
B158	Female	40	5	B384	Female	65	189
B163	Female	41	12	B385	Male	79	18
B165	Female	63	139	B386	Female	66	17
B172	Male	49	11	B387	Male	66	9
B208	Female	55	30	B392	Male	63	59
B216	Female	63	90	B391	Male	75	17
B219	Male	79	27	B393	Female	50	35
B221	Male	78	12	B395	Female	80	153
B369	Male	63	-	B401	Female	66	23
B371	Male	77	30	B402	Female	56	112
B373	Female	65	12	B403	Male	78	-
B374	Female	58	5	B410	Female	64	145
B375	Male	59	24	B411	Female	68	37
B376	Male	60	2	B443	Female	48	4

Supplementary Figure

Supplementary Fig. S1. sCD46 level in the paired serum and blister fluid from 3 BP patients. ELISA assay was used to determine the sCD46 level in serums and blister fluids from 3 BP patients and 3 healthy controls.

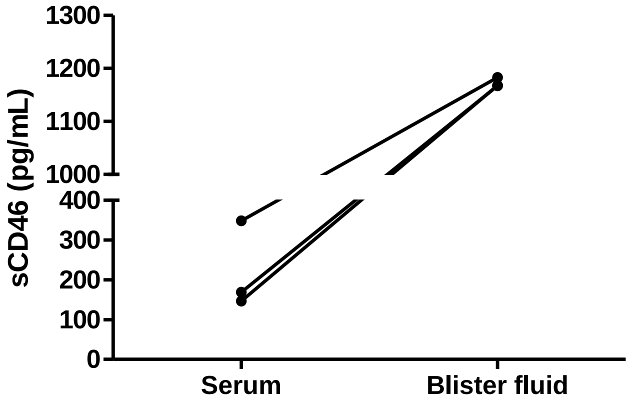

Supplementary Fig. S2. Small interference RNA targeting CD46 was transfected into Keratinocytes to detect the expression of CD46 protein. (A) Basal expression of CD46 in HaCaT human keratinocytes determined by immunofluorescence and **(B)** western blot. **(C)** The effectiveness of anti-CD46 siRNA relative to that of the negative control was determined by western blot after 48 h of transfection. **(D)** Effectiveness of the transfection system.

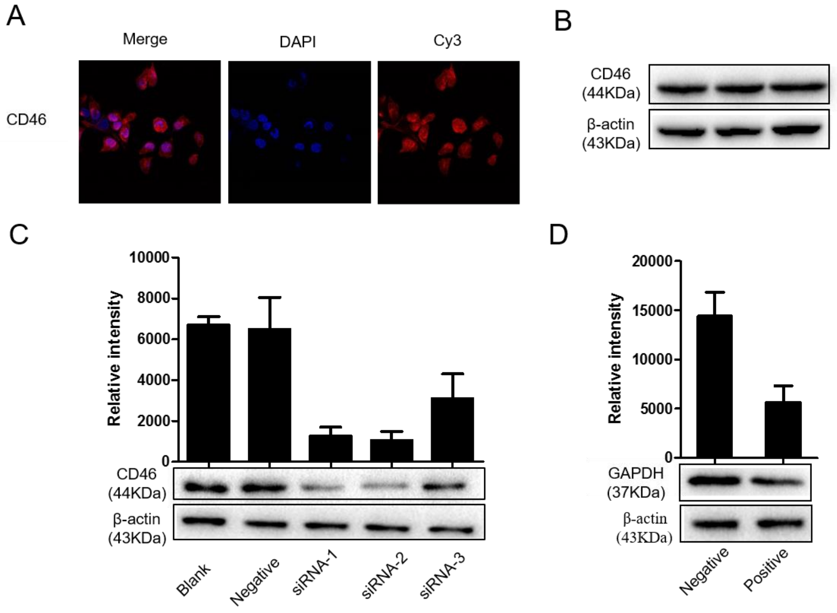
A
Merge
DAPI
Cy3
CD46
B
CD46 (44KDa)
β-actin (43KDa)
C
Relative intensity
10000
8000
6000
4000
2000
0
CD46 (44KDa)
β-actin (43KDa)
Blank
Negative
siRNA-1
siRNA-2
siRNA-3
D
Relative intensity
20000
15000
10000
5000
0
GAPDH (37KDa)
β-actin (43KDa)
Negative
Positive
